# Physical activity and depressive symptoms during pregnancy among Latina women: a prospective cohort study

**DOI:** 10.1186/s12884-018-1839-5

**Published:** 2018-06-20

**Authors:** Kathleen Szegda, Elizabeth R. Bertone-Johnson, Penelope Pekow, Sally Powers, Glenn Markenson, Nancy Dole, Lisa Chasan-Taber

**Affiliations:** 1 Department of Biostatistics & Epidemiology, School of Public Health & Health Sciences, University of Massachusetts, 414 Arnold House, 715 North Pleasant Street, Amherst, MA 01003-9304 USA; 2 Department of Psychological and Brain Sciences, University of Massachusetts, Amherst, MA USA; 3 Baystate Medical Center, Springfield, MA USA; 4 Carolina Population Center, University of North Carolina at Chapel Hill, Chapel Hill, NC USA; 5 Baystate Medical Center, Springfield, MA USA; 6 Public Health Institute of Western Massachusetts, Springfield, MA USA

**Keywords:** Physical activity, Antenatal depression, Antenatal depressive symptoms, Latina

## Abstract

**Background:**

Latina women are at increased risk for antenatal depressive disorders, which are common during pregnancy and are associated with elevated risk for poor maternal health and birth outcomes. Physical activity is a potential mechanism to reduce the likelihood of depressive symptoms. The purpose of the study was to assess whether total and domain-specific physical activity in early pregnancy reduced risk for elevated antenatal depressive symptoms in mid-late pregnancy in a population of Latina women at high-risk for depression.

**Methods:**

Data from 820 Latina participants in the prospective cohort study Proyecto Buena Salud was examined using multivariable logistic regression. Total, moderate/vigorous, and domain-specific physical activity (household/caregiving, occupational, sports/exercise, transportation) were assessed using the Pregnancy Physical Activity Questionnaire. The Edinburgh Postnatal Depression Scale was used to assess depressive symptoms and identify women with elevated symptoms indicative of at least probable minor depression and probable major depression.

**Results:**

A total of 25.9% of participants experienced at least probable minor depression and 19.1% probable major depression in mid-late pregnancy. After adjusting for important risk factors, no significant associations were observed between total physical activity (4th Quartile vs.1st Quartile OR = 1.02, 95% CI = 0.61, 1.71; p-trend = 0.62) or meeting exercise guidelines in pregnancy (OR = 0.96, 95% CI = 0.65, 1.41) and at least probable minor depression; similarly, associations were not observed between these measures and probable major depression. There was a suggestion of increased risk of probable major depression with high levels of household/caregiving activity (4th Quartile vs 1st Quartile OR = 1.51, 95% CI = 0.93, 2.46), but this was attenuated and remained not statistically significant after adjustment. When we repeated the analysis among women who did not have elevated depressive symptoms in early pregnancy (*n* = 596), findings were unchanged, though a nonsignificant protective effect was observed for sport/exercise activity and probable major depression in fully adjusted analysis (OR = 0.63, 95% CI = 0.30, 1.33).

**Conclusion:**

Among Latina women at high-risk for antenatal depression, early pregnancy physical activity was not associated with elevated depressive symptoms in mid-to-late pregnancy.

## Background

Depressive disorders affect up to an estimated 18% of women during pregnancy [1]. Depression relapse rates are particularly high during pregnancy, with some studies finding relapse rates as high as 43% [2]. Latina women in the United States are at increased risk for antenatal depression with some studies finding almost double the prevalence among Latina women compared to non-Latina White women [3]. Factors that likely contribute to this disparity include economic, acculturation, and social challenges experienced by Latinas in the U.S. [4], which increases their likelihood of depression overall, and thus the likelihood of reoccurrence or initial onset of a depressive episode during pregnancy. Because depression during pregnancy has been associated with increased risk of poor maternal health outcomes during and following pregnancy (e.g., pre-eclampsia, post-partum depression) [5, 6], as well as poor birth outcomes (e.g., admission to neonatal nurseries, small-for-gestational age) [7, 8], it is important to identify ways to prevent antenatal depression and reduce depressive symptoms in this at-risk population.

Physical activity may prevent the onset of depression [9], which is particularly important in populations that experience a high prevalence of depression and are at high-risk for antenatal depression. Exercise and physical activity are believed to prevent depression and reduce depressive symptoms by altering neurotransmitter and hormone levels that are associated with depression. It is also believed that exercise may reduce depression by distracting from depressive symptoms and promoting self-efficacy [10, 11]. Among women with antenatal depression, physical activity is an alternative option to antidepressant medications and psychotherapy, which are current common depression treatment options that may be underutilized or contraindicated. Studies suggest some antidepressants may negatively impact the developing fetus [12], which may result in hesitancy among patients to take these medications. Psychotherapy can be inaccessible and unaffordable, particularly among disadvantaged populations that experience socioeconomic challenges [13, 14]. Among Latinos, mental health services such as psychotherapy may be particularly underutilized because of cultural beliefs and concerns about stigma and fatalism [15]. Though current U.S. Health and Human Services guidelines recommend that pregnant women engage in at least 150 min of moderate intensity aerobic activity per week [16], women often do not meet these guidelines.

Studies conducted among women in the general population have generally found that physical activity is inversely associated with depression [17], but research in pregnant populations has been limited. Among studies conducted with pregnant women, findings have varied with some finding an inverse association [18–21], and others finding no association [22–24]. Among studies finding an inverse association, a number have been limited by their cross-sectional examination of physical activity and depression [18, 21], making it difficult to ascertain whether physical activity affected depression status or whether depression status lead to decreased physical activity levels. In addition, the majority of prior studies focused on exercise or leisure time activity and did not examine other domains of physical activity (i.e., household/caregiving and occupational activity), which may be an important consideration in populations that have limited ability to participate in leisure time activity. In the one study to evaluate other domains of activity, Demissie et al. found that among 1220 well educated, predominantly White women in North Carolina, higher levels of activity perceived as moderate to vigorous were associated with a reduction in depressive symptoms. However, perceived moderate to vigorous levels of some forms of household/caregiving activity increased risk for depressive symptoms [20]. As some household/caregiving activities are included in the list of activities recommended by the U.S. Health and Human Services as a means to achieve physical activity guidelines, it is important to understand the association between different types of activities and depression.

In a recent meta-analysis conducted by Daley et al. that examined randomized clinical trials conducted to assess the effect of exercise on the prevention and treatment of antenatal depression [14], the meta-analysis found a reduction in depressive symptoms among the six studies examined (standardized mean difference = − 0.46, 95% CI = − 0.87, − 0.05), but the authors noted the limited number and quality of studies available. The authors called for additional research, citing the need for more studies to better understand subgroup effects, including the effects of physical activity among nondepressed women, as only one study examined the protective effects of physical activity among nondepressed women at baseline [14].

Our study aimed to extend prior work by prospectively examining associations between total physical activity and domain-specific physical activity on elevated depressive symptoms during pregnancy in a Latina population at high-risk for antenatal depression. In addition, we examined physical activity and elevated depressive symptoms among nondepressed women to better understand whether physical activity may be associated with the onset of depression among women at high-risk.

## Methods

Data from Proyecto Buena Salud (PBS) was used to assess associations between physical activity and depressive symptoms. PBS was a prospective cohort study among Latina prenatal care patients conducted from 2006 to 2011 at Baystate Medical Center, a large tertiary care center in Western Massachusetts, which has approximately 4500 deliveries per year and serves an ethnically diverse population. Study design details have been published previously [25]. PBS was approved by the Institutional Review Boards at the University of Massachusetts, Amherst and Baystate Medical Center.

Women were recruited at prenatal care visits in early pregnancy (up to 20 weeks gestation). Participants were informed of study aims and procedures and asked to provide written informed consent. To reduce language and literacy barriers, interviews were conducted in English (73.7%) or Spanish by trained bilingual interviewers depending upon participant preference.

At the initial interview conducted at recruitment (mean = 12.4 weeks gestation), information was obtained on socio-demographic, acculturation, behavioral, physical activity, and depressive symptoms. Information on depressive symptoms was updated in mid-to-late pregnancy (mean = 25.7 weeks gestation). Information on medical history and clinical characteristics of the pregnancy were abstracted from the medical record after delivery.

### Study population

Eligibility for PBS was restricted to Latina women of Puerto Rican or Dominican Republic descent who were either born on one of these islands, or had at least one parent or two grandparents born on these islands. Other exclusion criteria included: multiple gestation; history of diabetes, hypertension, heart or chronic renal disease; less than 16 or greater than 40 years of age; and current use of medications thought to adversely affect glucose tolerance.

A total of 1579 women were enrolled into PBS. Women were excluded if they had a miscarriage (*n* = 68). Among eligible participants, a total of 1040 women had information on early pregnancy physical activity information. Among these women, 820 had information on mid-to-late pregnancy depressive symptoms and were included in the final dataset for analysis.

### Assessment of physical activity

Physical activity was assessed in early pregnancy using a modified version of the Pregnancy Physical Activity Questionnaire (PPAQ) [26]. The tool consists of a series of questions asking respondents to indicate intensity, frequency and time spent engaged in 35 activities from four domains: household/caregiving, occupational, sports/exercise, and transportation.

Average overall total early pregnancy physical activity weekly energy expenditure (metabolic equivalent [MET]-hrs/wk) was calculated by multiplying the amount of time spent on each activity by its intensity and then summing these values. Activity intensities were determined based on the Compendium of Physical Activities [27]; activities identified as having a different intensity during pregnancy were assigned a modified intensity value [28]. Total energy expenditure by domain (household/caregiving, occupational, sports/exercise, transportation) and intensity (moderate, vigorous) were also calculated. Moderate and vigorous activity were combined into a single category because there were so few women who engaged in vigorous activity. Total physical activity levels as well as those for moderate/vigorous, household, and transportation activity were categorized into quartiles. Because a large percentage of women did not engage in sports/exercise activity during pregnancy, women were categorized into none, sport/exercise levels “at or below the median,” and “above the median.” For occupational physical activity levels, women were categorized as unemployed, “at or below the median” and “above the median.”

Physical activity was also categorized as to whether participants met current U.S. Health and Human Services physical activity guidelines, which recommend that pregnant women engage in at least 150 min per week of moderate intensity aerobic physical activity [16]. Women were categorized as meeting the guidelines if they participated in > 7.5 MET-hrs per week of sports/exercise activities that were of moderate-intensity or greater (i.e., 30 min per day of activity at ≥ 3 METs multiplied by 5 days per week or 150 min). We focused on sports/exercise activity because studies suggest that household/caregiving or activities seen as burdensome may increase risk for depressive symptoms [29], whereas leisure time activity has most consistently found to reduce risk of depressive symptoms [17]. However, we also created a second variable for meeting guidelines that categorized women as meeting guidelines based on any domain of moderate or greater intensity activity.

The PPAQ has demonstrated good reliability and reasonable validity when compared to Actigraph accelerometer measures [26]. Intra-class correlations for two administrations of the PPAQ one week apart were 0.78, 0.82 and 0.81 for total, moderate and vigorous physical activity, and 0.83, 0.86 and 0.93 for sports/exercise, household/caregiving and occupational activity, respectively.

### Assessment of depression

Depressive symptoms were assessed in early and mid-to-late pregnancy with the Edinburgh Postnatal Depression Scale (EPDS) [30], which has been validated as a depression screening tool in pregnant and postpartum women [31]. The EPDS is a 10-item scale that asks respondents to indicate how frequently they have felt various mood states within the past seven days. Examples of items on the EPDS include, “I have been so unhappy that I have been crying,” and “Things have been getting on top of me.” Total scores range from 0 to 30. Women were categorized as to whether or not they had elevated depressive symptoms indicative of at least probable minor depression (scores13 or higher) or probable major depression (scores 15 or higher) [32]. To be consistent with the prior literature, EPDS scores were imputed for participants missing fewer than 10% of EPDS scale items by replacing the missing value with the participant’s average score of the nonmissing items [33].

The EPDS has been shown to have high sensitivity and specificity for major depression (sensitivity = 100%; specificity = 96%) and reasonable sensitivity and specificity for at least probable minor depression (sensitivity = 57%; specificity = 98%) using these cut-points in an English-speaking population [34]. The EPDS has also performed well as a depression screening tool in Spanish-speaking populations with good sensitivity and specificity for at least minor depression (sensitivity = 79%; specificity = 96%) and major depression (sensitivity = 83%; specificity = 97%) postnatally [35].

### Covariates

Information on maternal age, education level, income, whether the participant was living with a partner, generation in the continental U.S., and overall acculturation (Psychological Acculturation Scale) [36] was obtained at the initial pregnancy interview. Smoking status and alcohol consumption were assessed at each pregnancy interview. Psychosocial stress was assessed by Cohen’s Perceived Stress Scale [37] and anxiety was assessed via the State-Trait Anxiety Survey [38]. Pre-pregnancy weight, height, parity and presence of morning sickness during the pregnancy were obtained through the medical record. Body mass index (BMI) was calculated as weight (kg)/height (m^2^).

### Data analysis

Univariate statistics were used to describe the study population. Baseline characteristics were compared between women with each of the respective probable major depression measures and women who did not have elevated depressive symptom scores. Unadjusted and multivariable logistic regression analyses were used to assess associations between physical activity in early pregnancy and at least probable minor depression and probable major depression in mid-to-late pregnancy.

We examined associations between total physical activity, moderate/vigorous physical activity, domain-specific physical activity (household/caregiving, occupational, sports/exercise, transportation), and whether women met U.S. Health and Human Services (HHS) physical activity guidelines, and each of the elevated depressive symptom outcome measures in separate models. Maternal age, education, living with a partner/spouse and depression in early pregnancy (assessed at baseline via the EPDS) were included as a priori confounders in models because they have been identified as risk factors for depression, or in the case of living with a partner/spouse, as a proxy for a strong protective factor (social support) [13, 39, 40]. Weeks between early and mid-to-late pregnancy interviews was also included in multivariable models in addition to any other potential confounders that changed the odds ratio for the depression measure by more than 10% with their inclusion in the model. Additional covariates identified for inclusion through this method included parity and generation in the U.S. For domain-specific multivariable models, we additionally adjusted for MET-hrs/wk. from all other domains of physical activity.

We chose not to include stress and anxiety in our multivariable models because they were both highly correlated with depression (*r* = 0.61–0.78) and we were interested in assessing associations for all cases of depression and their inclusion would have obscured associations when anxiety/stress and depression co-occurred. In a sensitivity analysis, we repeated analyses using U.S. HHS physical activity guidelines defined as moderate/vigorous physical activity from all domains of activity in early pregnancy and mid-to-late pregnancy.

Because the effects of physical activity on subsequent depression may vary by baseline depression status [41], we then repeated the above analyses among women who did not have elevated depressive symptoms in early pregnancy (i.e. did not have at least probable minor depression) (*n* = 596) [41]. Finally, we examined differences in characteristics between women with and without information on mid-to-late pregnancy depressive symptom to assess potential effects of missing data. All analyses were conducted using SAS version 9.2 (SAS Institute Inc., Cary, NC).

## Results

Among study participants, 25.9% of women had elevated depressive symptoms indicative of at least probable minor depression and 19.1% had depressive symptoms indicative of probable major depression in mid-to-late pregnancy. The mean age was 21.6 years (SD = 4.9). Women were generally of low socioeconomic status with almost half (48.7%) reporting that they did not complete high school and only 6.1% of women reporting an annual household income of $30,000 or greater (Table 1). Almost half of participants (48.4%) were born in Puerto Rico or the Dominican Republic and over two thirds of the study population had low levels of acculturation (80.7%). Women who smoked during early pregnancy were more likely to have at least probable minor depression and probable major depression in mid-to-late pregnancy. In addition, women who were obese (BMI ≥ 30 kg/m^2^) or who did not live with a partner/spouse were more likely to have at least probable minor depression in mid-to-late pregnancy; women who had higher levels of parity were more likely to have probable major depression in mid-to-late pregnancy.

**Table 1:** Participant Characteristics by Elevated Depressive Symptom Scores in Mid-to-Late Pregnancy: Proyecto Buena Salud, 2006–2011

	Total Population		At Least Probable Minor Depression (EPDS ≥13)			Probable Major Depression (EPDS ≥15)		
	n^a^	%	cases	%	*p*-value^b^	cases	%	*p*-value^b^
Maternal Age								
16–19	270	32.9	61	28.8	0.40	42	26.9	0.25
20–24	315	38.4	83	39.2		62	39.7	
25–29	144	17.6	40	18.9		30	19.2	
≥ 30	91	11.1	28	13.2		22	14.1	
Education								
Less than high school	396	48.8	111	52.9	0.37	87	56.1	0.09
High school graduate or GED	270	33.3	63	30.0		41	26.5	
Post high school	146	18.0	36	17.1		27	17.4	
Income								
less than $15,000	242	30.1	75	36.1	0.15	58	37.4	0.13
$15,000–$30,000	121	15.0	27	13.0		18	11.6	
$30,000 or greater	49	6.1	10	4.8		8	5.2	
Don’t Know/Refused	393	48.8	96	46.2		71	45.8	
Acculturation								
Low	635	80.7	161	79.7	0.68	121	80.7	0.99
High	152	19.3	41	20.3		29	19.3	
Generation in U.S.								
Born in PR/DR	382	48.4	99	49.0	0.72	74	49.7	0.11
Parent born in PR/DR	363	46.0	94	46.5		72	48.3	
Grandparent born in PR/DR	44	5.6	9	4.5		3	2.0	
Live with partner/spouse								
no	405	50.3	117	56.0	0.05	82	52.9	0.46
yes	401	49.8	92	44.0		73	47.1	
Pre-Pregnancy BMI								
less than 18.5	43	5.3	11	5.3	< 0.01	7	4.6	0.22
18.5- < 25.0	395	48.8	109	52.4		78	51.3	
25.0- < 30.0	180	22.3	29	13.9		25	16.5	
30 or greater	191	23.6	59	28.4		42	27.6	
Parity								
0 live births	336	41.5	75	36.1	0.12	50	32.9	0.03
1 live birth	255	31.5	67	32.2		50	32.9	
2 or more live births	219	27.0	66	31.7		52	34.2	
Smoking (early pregnancy)								
no	672	86.6	162	79.0	< 0.01	117	77.0	< 0.01
yes	104	13.4	43	21.0		35	23.0	
Alcohol consumption (early pregnancy)								
no	756	97.4	197	26.1	0.06	147	19.4	0.24
yes	20	2.6	9	45.0		6	30.0	

^a^Numbers may not total to 820 due to missing data

^b^*P*-values were calculated using chi-square tests and are for comparisons between cases and noncases

On average, household/caregiving physical activity accounted for the largest average proportion of energy expenditure (56.0%) and sports/exercise activity the least (6.4%). Almost half of women did not engage in sports/exercise activity in early pregnancy (46.5%) and 51.7% of women were not employed in early pregnancy.

We examined associations between total physical activity in early pregnancy and elevated depressive symptoms in mid-to-late pregnancy (Table 2). In unadjusted analyses, the odds ratio for at least probable minor depression for women with the highest level of total physical activity as compared to the lowest level was elevated, but not statistically significant (4th Quartile [Q_4_] vs. 1st Quartile [Q_1_]_:_ odds ratio [OR] = 1.28, 95% confidence interval [CI] = 0.83, 1.98; p-trend = 0.21). After adjustment for age, education, living situation, parity, generation in the U.S., at least probable minor depression in early pregnancy, and weeks between interviews, this odds ratio was attenuated to 1.02, (95% CI = 0.61, 1.71; p-trend = 0.62). Results were similar for probable major depression (unadjusted Q_4_ vs. Q_1:_ OR = 1.28, 95% CI = 0.79, 2.05; p-trend = 0.27 vs. adjusted Q_4_ vs. Q_1:_ OR = 0.96, 95% CI = 0.55, 1.70; p-trend = 0.83). Similarly, we did not observe significant associations between moderate/vigorous activity or meeting physical activity guidelines and mid-to-late pregnancy elevated depressive symptom outcomes. We also did not observe significant associations in a sensitivity analysis using U.S. HHS physical activity guidelines defined as moderate/vigorous physical activity from all domains of activity.

**Table 2:** Odds ratios of mid-to-late pregnancy probable depression by various early pregnancy physical activity levels: Proyecto Buena Salud, 2006–2011

	At least Probable Minor Depression (EPDS≥13)								Probable Major Depression (EPDS≥15)							
	Cases		Unadjusted		Adjusted^b^		Adjusted^c^		Cases		Unadjusted		Adjusted^b^		Adjusted^c^	
	n	%	OR	95% CI	OR	95% CI	OR	95% CI	n	%	OR	95% CI	OR	95% CI	OR	95% CI
Total physical activity																
1st Quartile	53	27.9	referent		referent				40	21.1	referent		referent			
2nd Quartile	36	19.8	0.64	0.39, 1.03	0.52	0.30, 0.90	n/a		27	14.8	0.65	0.38, 1.12	0.55	0.30, 1.02	n/a	
3rd Quartile	44	22.1	0.73	0.46, 1.16	0.72	0.42, 1.23			32	16.1	0.72	0.43, 1.20	0.72	0.39, 1.31		
4th Quartile	64	32.5	1.28	0.83, 1.98	1.02	0.61, 1.71			49	25.4	1.28	0.79, 2.05	0.96	0.55, 1.70		
p-trend			0.21		0.62						0.27		0.83			
Met exercise guidelines^a^																
no	142	25.7	referent		referent				101	18.3	referent		referent			
yes	68	26.6	1.05	0.75, 1.46	0.96	0.65, 1.41	n/a		53	20.7	1.17	0.80, 1.69	1.12	0.73, 1.71	n/a	
Moderate/vigorous intensity																
1st Quartile	51	25.6	referent		referent				34	17.1	referent		referent			
2nd Quartile	44	21.8	0.81	0.51, 1.28	0.60	0.35, 1.03	n/a		36	17.8	1.05	0.63, 1.76	0.78	0.43, 1.43	n/a	
3rd Quartile	57	27.4	1.10	0.71, 1.70	0.73	0.44, 1.22			42	20.2	1.23	0.74, 2.03	0.86	0.48, 1.53		
4th Quartile	58	28.7	1.17	0.75, 1.82	0.89	0.53, 1.50			42	20.8	1.27	0.77, 2.10	0.94	0.52, 1.69		
p-trend			0.28		0.92						0.28		0.95			
Domain of Activity																
Household/ caregiving																
1st Quartile	49	26.6	referent		referent		referent		34	18.5	referent		referent		referent	
2nd Quartile	48	24.5	0.89	0.56, 1.42	1.11	0.64, 1.93	1.03	0.59, 1.81	39	19.9	1.10	0.66, 1.83	1.37	0.73, 2.55	1.26	0.67, 2.37
3rd Quartile	44	21.8	0.77	0.48, 1.22	0.67	0.38, 1.18	0.64	0.36, 1.13	27	13.4	0.68	0.39, 1.18	0.59	0.30, 1.14	0.56	0.29, 1.10
4th Quartile	60	30.0	1.18	0.76, 1.84	0.93	0.53, 1.64	0.85	0.48, 1.52	51	25.5	1.51	0.93, 2.46	1.18	0.63, 2.21	1.09	0.57, 2.08
p-trend			0.58		0.45		0.31				0.24		0.80		0.68	
Occupational																
unemployed	115	29.3	referent				referent		86	21.9	referent		referent		referent	
at or below median	42	19.9	0.60	0.40, 0.90	0.74	0.47, 1.17	0.78	0.48, 1.26	31	14.7	0.62	0.39, 0.96	0.84	0.50, 1.42	0.96	0.56, 1.63
above median	52	25.7	0.84	0.57, 1.23	0.88	0.55, 1.39	0.85	0.53, 1.37	37	18.3	0.80	0.52, 1.23	0.85	0.51, 1.41	0.85	0.50, 1.44
Sports/Exercise																
none	94	25.0	referent		referent		referent		67	17.8	referent		referent		referent	
at or below median	59	27.8	1.16	0.79, 1.69	1.27	0.82, 1.97	1.1	0.70, 1.75	43	20.3	1.17	0.77, 1.80	1.33	0.81, 2.18	1.16	0.69, 1.94
above median	57	25.9	1.05	0.72, 1.54	0.98	0.63, 1.52	0.9	0.57, 1.44	44	20.0	1.15	0.76, 1.76	1.13	0.70, 1.83	0.98	0.59, 1.64
Transportation																
1st Quartile	61	26.2	referent		referent		referent		47	20.2	referent		referent		referent	
2nd Quartile	41	24.3	0.90	0.57, 1.43	0.82	0.48, 1.41	0.74	0.42, 1.30	26	15.4	0.72	0.43, 1.22	0.64	0.35, 1.19	0.55	0.29, 1.06
3rd Quartile	46	22.9	0.84	0.54, 1.30	0.99	0.60, 1.65	0.95	0.56, 1.62	34	16.9	0.81	0.49, 1.31	0.94	0.53, 1.65	0.82	0.45, 1.48
4th Quartile	62	30.1	1.21	0.80, 1.84	1.21	0.75, 1.97	1.22	0.72, 2.06	48	23.3	1.20	0.76, 1.90	1.10	0.65, 1.88	1.06	0.60, 1.90
p-trend			0.47		0.36		0.39				0.43		0.52		0.68	

^a^Met American College of Obstetricians and Gynecologists guidelines of > 7.5 MET-hrs/week in sports/exercise activities of moderate-intensity or greater

^b^Adjusted for age, education, live with partner or spouse, parity, generation in U.S., at least probable minor depression in early pregnancy and weeks between interviews

^c^Additionally adjusted for energy expenditure from other domains of physical activity

We then examined associations between domains of early pregnancy physical activity and mid-to-late pregnancy elevated depressive symptom outcomes (Table 2). Women with the highest level of household/caregiving activity had 1.51 times the odds of probable major depression compared to women with the lowest level (95% CI = 0.93, 2.46), but again this was not statistically significant and was further attenuated after adjustment for important risk factors (OR = 1.18, 95% CI = 0.63, 2.21). Point estimates for the other domains of physical activity (i.e., occupational, sports/exercise, and transportation) and odds ratios of at least probable minor depression or probable major depression were closer to 1.0 and also not statistically significant in both unadjusted and adjusted analyses (Table 2). In domain-specific analyses that included additional adjustment for MET-hrs/wk. from other domains of physical activity, we similarly observed no significant associations.

We then repeated the above analyses restricted to women who did not have elevated depressive symptoms (i.e. did not have at least probable minor depression) in early pregnancy (*n* = 596) (Table 3). Similar to our primary analysis, we did not observe significant associations between any of the physical activity measures and depressive symptom outcomes in unadjusted or adjusted analyses. For example, for total activity the adjusted odds ratio for probable major depression when comparing Q_4_ to Q_1_ was 1.21 (95% CI = 0.56, 2.64; p-trend = 0.70) and for meeting exercise guidelines was 0.82 (95% CI = 0.43, 1.55) when compared to women who did not meet guidelines. Women with the highest levels of sports/exercise activity who did not have elevated depressive symptoms in early pregnancy appeared to have a lower odds of probable major depression (OR 0.63, 95% CI = 0.30, 1.33) after adjusting for important risk factors and other domains of activity, but this was not statistically significant.

**Table 3:** Odds ratios of mid-to-late pregnancy probable depression by early pregnancy physical activity levels among nondepressed women in early pregnancy: Proyecto Buena Salud, 2006–2011

	At least Probable Minor Depression (EPDS ≥13)								Probable Major Depression (EPDS ≥15)							
	Cases		Unadjusted		Adjusted^b^		Adjusted^c^		Cases		Unadjusted		Adjusted^b^		Adjusted^c^	
	n	%	OR	95% CI	OR	95% CI	OR	95% CI	n	%	OR	95% CI	OR	95% CI	OR	95% CI
Total physical activity																
1st Quartile	24	17.1	referent		referent				16	11.4	referent		referent			
2nd Quartile	17	12.8	0.71	0.36, 1.39	0.66	0.33, 1.32	n/a		10	7.5	0.63	0.28, 1.44	0.57	0.24, 1.37	n/a	
3rd Quartile	19	12.6	0.70	0.36, 1.34	0.71	0.36, 1.43			12	8.0	0.67	0.31, 1.47	0.70	0.30, 1.62		
4th Quartile	22	17.3	1.01	0.54, 1.91	1.04	0.53, 2.03			16	12.6	1.12	0.53, 2.34	1.21	0.56, 2.64		
p-trend			0.96		0.94						0.79		0.70			
Met exercise guidelines^a^																
no	69	16.7	referent		referent		n/a		43	10.4	referent		referent		n/a	
yes	20	11.6	0.65	0.38, 1.11	0.69	0.40, 1.18			14	8.1	0.76	0.40, 1.42	0.82	0.43, 1.55		
Moderate/vigorous intensity																
1st Quartile	25	16.0	referent		referent		n/a		15	9.6	referent		referent		n/a	
2nd Quartile	23	15.2	0.94	0.51, 1.74	0.93	0.49, 1.77			16	10.6	1.11	0.53, 2.34	1.11	0.51, 2.41		
3rd Quartile	20	14.4	0.88	0.47, 1.67	0.92	0.47, 1.79			11	7.9	0.81	0.36, 1.82	0.90	0.39, 2.08		
4th Quartile	21	14.7	0.90	0.48, 1.70	0.90	0.46, 1.76			15	10.5	1.10	0.52, 2.34	1.14	0.51, 2.55		
p-trend			0.71		0.76						0.99		0.88			
Domain of Activity																
Household/ caregiving																
1st Quartile	25	18.3	referent		referent		referent		15	11.0	referent		referent		referent	
2nd Quartile	22	14.4	0.75	0.40, 1.41	0.94	0.48, 1.86	0.95	0.48, 1.91	16	10.5	0.95	0.45, 2.00	1.25	0.55, 2.85	1.20	0.52, 2.77
3rd Quartile	17	11.6	0.59	0.30, 1.15	0.67	0.32, 1.40	0.71	0.33, 1.50	8	5.5	0.47	0.19, 1.15	0.58	0.22, 1.56	0.57	0.21, 1.55
4th Quartile	21	15.9	0.85	0.45, 1.60	0.99	0.47, 2.06	0.98	0.46, 2.11	17	12.9	1.20	0.57, 2.52	1.60	0.67, 3.84	1.53	0.62, 3.78
p-trend			0.46		0.75		0.78				0.99		0.55		0.61	
Occupational																
unemployed	44	16.5	referent		referent		referent		28	10.5	referent		referent		referent	
at or below median	20	11.8	0.68	0.38, 1.20	0.58	0.32, 1.07	0.58	0.31, 1.09	13	7.7	0.71	0.36, 1.41	0.65	0.31, 1.37	0.68	0.32, 1.45
above median	23	15.5	0.93	0.54, 1.61	0.80	0.42, 1.53	0.72	0.37, 1.43	15	10.1	0.96	0.49, 1.86	0.77	0.35, 1.73	0.67	0.29, 1.56
Sports/Exercise																
none	45	16.0	referent		referent		referent		31	11.0	referent		referent		referent	
at or below median	26	16.7	1.05	0.62, 1.78	1.08	0.63, 1.87	0.93	0.52, 1.67	13	8.3	0.73	0.37, 1.45	0.80	0.40, 1.61	0.58	0.27, 1.24
above median	18	12.1	0.72	0.40, 1.30	0.76	0.41, 1.38	0.65	0.34, 1.42	13	8.7	0.77	0.39, 1.52	0.85	0.42, 1.71	0.63	0.30, 1.33
Transportation																
1st Quartile	27	15.7	referent		referent		referent		16	9.3	referent		referent		referent	
2nd Quartile	18	14.8	0.93	0.49, 1.78	0.87	0.44, 1.73	0.76	0.36, 1.57	12	9.8	1.06	0.48, 2.34	0.98	0.42, 2.26	0.88	0.37, 2.09
3rd Quartile	26	16.8	1.08	0.60, 1.95	1.11	0.59, 2.08	1.01	0.53, 1.95	18	11.6	1.28	0.63, 2.61	1.34	0.62, 2.86	1.05	0.47, 2.32
4th Quartile	18	13.0	0.80	0.42, 1.52	0.90	0.46, 1.75	0.76	0.36, 1.58	11	7.9	0.84	0.38, 1.87	0.94	0.41, 2.17	0.72	0.29, 1.80
p-trend			0.65		0.95		0.63				0.89		0.91		0.61	

^a^Met American College of Obstetricians and Gynecologists guidelines of > 7.5 MET-hrs/week in sports/exercise activities of moderate-intensity or greater

^b^Adjusted for age, education, live with partner or spouse, parity, generation in U.S., and weeks between interviews

^c^Additionally adjusted for energy expenditure from other domains of physical activity

Lastly, women who were missing mid-to-late pregnancy depressive symptom information did not differ from women with this information by early pregnancy probable depression status (at least probable minor depression or probable major depression) and also did not differ according to age, BMI, parity, generation in the U.S., whether they lived with a partner or spouse, or early pregnancy smoking status; however they had higher levels of education (*p* < 0.01), income (*p* = 0.01), and acculturation (*p* = 0.02) and were more likely to have had a preterm birth (*p* < 0.001).

## Discussion

In our prospective cohort study of 820 Latina women, we did not observe statistically significant associations between total and domain-specific physical activity in early pregnancy and mid-to-late pregnancy elevated depressive symptoms. We similarly did not observe significant associations when restricting our analyses to women who did not have elevated depressive symptoms in early pregnancy.

Few studies have examined the association between total and domain specific physical activity and depression during pregnancy. Our findings that physical activity and sports/exercise activity were not associated with elevated depressive symptom outcomes are consistent with the findings of several other studies examining these associations [22–24]. Similar to our findings, Symons-Downs et al. did not find an independent association between exercise behavior and depressive symptoms in prospective analysis during pregnancy in their study of 230 predominantly White, highly educated women [22]. In the one study to evaluate other domains of activity, Demissie et al. similarly did not find significant associations between absolute levels (MET-hrs per week) of total (OR = 0.71, 95% CI = 0.42, 1.18) or domain-specific moderate to vigorous physical activity and elevated depressive symptoms among 1220 predominantly White women in North Carolina [20]. However, when the authors used participant perception of intensity to classify activities, they found that moderate levels of total moderate to vigorous activity was inversely associated with elevated depressive symptoms (OR = 0.56, 95% CI = 0.38, 0.83) and that moderate levels of moderate to vigorous household activity increased risk for elevated depressive symptoms, suggesting that perception of physical activity may impact the association.

Differences in findings may be due to differences in study populations, as well as, differential effects of different types of physical activity. For example, women in our study were predominantly of low socioeconomic status and had high levels of baseline depressive symptoms, which may impact associations as physical activity has been suggested to have different effects depending on levels of depression [41]. Importantly, the largest source of physical activity energy expenditure in our population was household/caregiving with almost half of women not participating in any sports/exercise activity. Studies suggest that type of physical activity has differential effects on depression in the general population, with exercise/leisure time activity generally having a positive effect on depression and household/caregiving physical activity either having no effect or increasing risk for depressive symptoms [17, 29]. Household/caregiving activity may be stressful for women, which could negate any positive effects of physical activity or increase risk for depressive symptoms. Indeed, a study by Molarius et al. found that the more burdensome study participants perceived domestic activities, the greater the risk for depressive symptoms [29].

A number of studies have examined associations between physical activity and antenatal depression among patients who were not diagnosed as clinically depressed at baseline [14, 42], however, antenatal depression is often undiagnosed [43] and no studies that we are aware of excluded women who had elevated depressive symptoms indicative of probable depression at baseline. In the recent meta-analysis by Daley et al., the authors identified one randomized controlled trial of an aerobic exercise program conducted among clinically nondepressed patients by Robledo-Colonia et al. which found an average four-point reduction in depressive symptom score among intervention group participants [14, 44]. However, some participants had baseline depressive symptom scores, as measured by the Center for Epidemiologic Studies - Depression Scale, which were higher than the widely used screening cut-point for significant depressive symptoms or depression [44, 45], suggesting that some women may have had undiagnosed depression at baseline. In our analysis, after excluding women with baseline depressive symptoms, sports/exercise may have had a protective impact on incident probable major depression, but this was not statistically significant.

Our study had several potential limitations. First, the EPDS is a tool that assesses depressive symptoms and is not a clinical measure of depression. However, the EPDS is widely used to indicate probable depressive disorder and has been demonstrated to have strong sensitivity and specificity for depression during pregnancy when validated compared to a structured clinical interview [34]. Though the validation studies for antenatal depression were conducted using the English version of the tool and the Spanish version has not been validated, we expect the Spanish version to have relatively comparable sensitivity and specificity because both the Spanish and English versions had good sensitivity and specificity for postnatal depression using the same cut-offs to define depression [34, 35]. If bias did occur as a result of misclassification of depression, results would have been biased towards the null. However, the EPDS minimizes some other potential forms of bias because unlike other depression screening tools that have been used in many of the studies that have examined associations between physical activity and elevated depressive symptoms during pregnancy, the EPDS takes into account common somatic depressive symptoms that are also symptoms of pregnancy.

We had information on a number of important risk factors that may confound associations between physical activity and depression during pregnancy, however, we did not have information on history of pre-pregnancy depression, which is a risk factor for antenatal depression. Nonetheless, as depression relapse rates are high during pregnancy [2], we anticipate that many of these women would have a recurrence during pregnancy. By adjusting for early pregnancy depression, we were able to adjust for this confounding to some degree, though there are some women who may have had a recurrence later in pregnancy, which could lead to confounding if history of depression was associated with physical activity. In addition, we did not have information on diet, another potential confounder. Exercise behavior is positively associated with healthy eating [46] and studies suggest that nutrient deficiency (e.g. n-3 fatty acids, B vitamins, folate) and high energy intake (particularly that of unhealthy food) may be associated with depression [47, 48]. In addition, diet may be on the causal pathway between physical activity and depression because physical activity may suppress appetite and studies suggest caloric restriction may have an anti-depressant effect [49].

It is possible that women who were depressed in early pregnancy were less likely to complete the mid-to-late pregnancy interview on depression. We found that women missing mid-to-late pregnancy depressive symptom information did not differ by early pregnancy probable depression status or on the majority of sociodemographic, medical history, and behavioral factors than women not missing this information, although they had higher levels of education, income, and acculturation, and were more likely to have had a preterm birth. Socioeconomic status, which includes factors such as income and education, is positively associated with exercise activity [50, 51], and inversely associated with depression [52] with individuals of lower socioeconomic status experiencing higher rates of depression. Loss of women with higher incomes and levels of education during follow-up would have potentially biased a protective finding of sports/exercise activity towards the null. In addition, as a result of this loss to follow-up, we had lower power to detect effects than originally planned and the study needs replication in a larger sample. Finally, we used odds ratios as our estimates of effect. Though they are valid estimates of effect, caution should be taken in inferring risk as they may overestimate effects because the outcomes were not rare [53].

## Conclusion

Physical activity has been suggested as a potential mechanism to prevent the onset of antenatal depression and/or alleviate depressive symptoms. We found that total and domain-specific physical activity were not associated with elevated depressive symptom outcomes in mid- to late pregnancy in an at-risk population of Latina women with a high prevalence of elevated symptoms indicative of probable depression outcomes.

## Abbreviations

95% CI: 95% confidence interval
BMI: Body mass index
EPDS: Edinburgh Postnatal Depression Scale
HHS: Health and human services
MET: Metabolic equivalent
OR: Odds ratio
PBS: Proyecto Buena Salud
PPAQ: Pregnancy physical activity questionnaire
Q1: 1st quartile
Q4: 4th quartile
